# Acaricidal efficacy of ultraviolet-C irradiation of *Tetranychus urticae* adults and eggs using a pulsed krypton fluoride excimer laser

**DOI:** 10.1186/s13071-021-05085-7

**Published:** 2021-11-17

**Authors:** Jean-Luc Gala, Ott Rebane, Jérôme Ambroise, Sergey Babichenko, Omar Nyabi, Thierry Hance

**Affiliations:** 1 Centre for Applied Molecular Technologies, Institute of Clinical and Experimental Research, Université catholique de Louvain, Tour Claude Bernard, Avenue Hippocrate 54-55, First floor, B1.54.01, 1200 Brussels, Belgium; 2 LDI Innovation OÜ, Sära 7, Peetri, Estonia; 3 Biodiversity Research Centre, Earth and Life Institute, Université catholique de Louvain, Croix du sud 4-5, 1348 Louvain-la-Neuve, Belgium

**Keywords:** Germicidal effect, Physical control, Ultraviolet-C, Pulsed irradiation, Excimer laser, Krypton fluoride, *Tetranychus urticae*, Mortality, Egg hatchability

## Abstract

**Background:**

Pulsed ultraviolet (UV)-C light sources, such as excimer lasers, are used in emerging non-thermal food-decontamination methods and also have high potential for use in a wide range of microbial decontamination applications. The acaricidal effect of an experimental UV-C irradiation device was assessed using female adults and eggs of a model organism, the two-spotted spider mite *Tetranychus urticae*.

**Methods:**

UV-C light was generated by a pulsed krypton fluoride excimer laser operating at 248-nm emission wavelength. The pulse energy and pulse repetition rate were 5 mJ and up to 100 Hz, respectively. The distance from the light source to the target was 150 mm; the target surface area was 2.16 cm^2^. The exposure time for the mites and fresh eggs varied from 1 to 4 min at 5–300 mW, which corresponded to UV doses of 5–80 kJ/m^2^. Post-irradiation acaricidal effects (mite mortality) were assessed immediately and also measured at 24 h. The effects of UV-C irradiation on the hatchability of eggs were observed daily for up to 12 days post-irradiation.

**Results:**

The mortality of mites at 5 and 40 kJ/m^2^ was 26% and 92%, respectively. Mite mortality reached 98% at 80 kJ/m^2^. The effect of exposure duration on mortality was minimal. The effect of irradiation on egg hatchability was even more significant than that on adult mite mortality, i.e. about 100% egg mortality at an accumulated dose of as little as 5 kJ/m^2^ for each exposure time.

**Conclusions:**

A high rate of mite mortality and lethal egg damage were observed after less than 1 min of exposure to 5 mJ UV-C pulsed irradiation at 60 Hz. Pending further developments (such as beam steering, beam shaping and miniaturisation) and feasibility studies (such as testing with mites in real-life situations), the reported results and characteristics of the UV-C generator (modulation of energy output and adaptability to varying spot sizes) open up the use of this technology for a vast field of acaricidal applications that require long-range radiation.

**Graphical Abstract:**

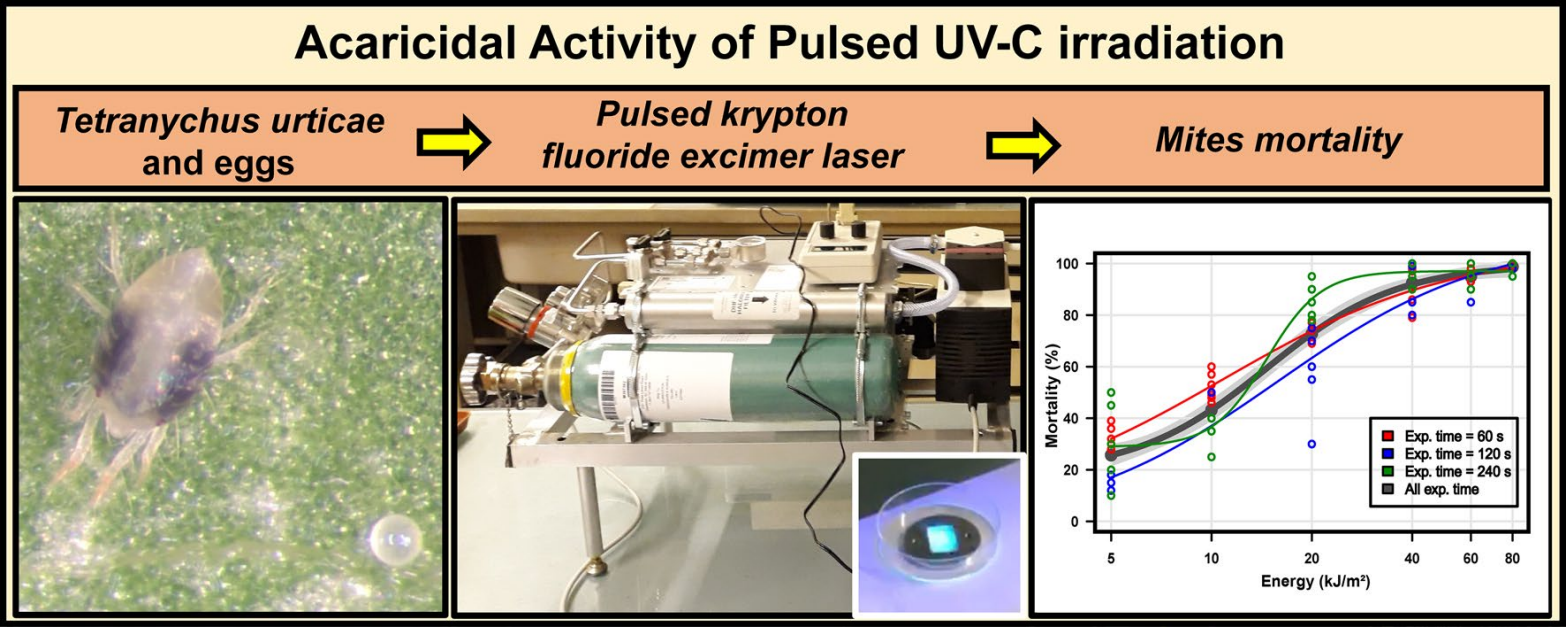

**Supplementary Information:**

The online version contains supplementary material available at 10.1186/s13071-021-05085-7.

## Background

New control and decontamination strategies based on physical technologies are promising means of improving microbial safety. The aims of these strategies include improvement of the control of foodborne pathogens [1, 2], the inactivation of food and environmental biowarfare agents [3–5], and arthropod pest control [6]. One of the main advantages of microbicidal non-chemical inactivation procedures is that they are considered eco-friendly as they do not leave potentially toxic residues in the environment.

Non-ionising UV-C irradiation, alone or in combination with other cleaning and disinfection methods, is a physical technology that is increasingly used for a wide range of applications. It is used in the food industry and for food safety [7, 8], in agriculture [9], for water decontamination [10, 11], for indoor air purification and surface cleaning [12–14], in healthcare environments [15–19], and for the treatment of personal protective equipment [20]. The latter application is under particular scrutiny at the moment in the context of the coronavirus disease pandemic and related critical shortages in the supply chain of personal protective equipment [21, 22].

Germicidal ultraviolet irradiation (GUVI) encompasses a continuous range of wavelengths in the UV region between 200 and 280 nm. Different types of lamps can be used for GUVI, as reviewed by Bergman [23] and the IES Photobiology Committee [24]. The most common type of these lamps is the low-pressure mercury lamp, which emits mainly at 254 nm and has been used for decades to disinfect both room air and surfaces. Pulsed xenon lamps emit short pulses (of the order of milliseconds) of broad-spectrum radiation including wavelengths in the UV and near-infrared range. Pulsed light technology involves the application of intense light in the form of short pulses of high intensity on the target of interest, with high penetration, maximum power emission capacity, and peak power distribution during the short pulses. Pulsed light has been shown to effectively inactivate microorganisms such as bacteria, yeasts, molds and viruses [25]. As a result, this technology is an emerging non-thermal food processing method used to decontaminate food products, food contact surfaces and packaging; however, due to the hazards associated with UV radiation, its use is not compatible with continuous human presence. In contrast, excimer lamps are quasi-monochromatic sources that emit over a wide range of the UV region, depending on the gas used. Krypton fluoride (KrF) excimer lamps emit significantly in the UV-C region in a narrow band of wavelengths around 222 nm [full width at half maximum (FWHM) ~ 3 nm]. According to the corresponding safety standards, at this wavelength, the effect of UV radiation on human skin is slightly reduced, which increases the possibility of using GUVI in areas where people are present as the allowed exposure limit is almost tenfold that for light at 270 nm (https://uv-light.co.uk/what-are-the-exposure-limit-values-elvs-for-uv-light). UV-C light-emitting diodes (LEDs) produce a narrow (FWHM ~ 10 nm) wavelength band of UV radiation with peak wavelengths at 266 nm, 270 nm, and 275 nm, etc. [26]. Despite the advantages of their small size, high efficiency and long lifetime, LED sources can not be used to focus light on a target over a long distance due to their large beam divergence in relation to that of lasers.

In the work reported here, we assessed the GUVI of an experimental pulsed UV-C light source based on a pulsed KrF excimer laser which operates at 248 nm emission wavelength; it has a pulse energy > 5 mJ and a pulse repetition rate of up to 100 Hz. At laser output, the laser radiation has a maximum power of 500 mW, which results in a power density of 83 kW/m^2^. The model organism used was the two-spotted spider mite *Tetranychus urticae*. This mite is a worldwide pest of many agricultural crops, including fruit, vegetables and ornamentals, and many agronomic crops [27]. This model organism has also been used to assess the killing efficacy of radiation from a broad-spectrum white LED (420–680 nm) and blue LED (460 nm) [28] in comparison with that from a commercial UV-C lamp with a peak UV output of ~ 254 nm [29, 30]. In the present study, the immediate post-irradiation germicidal effects (mite mortality and egg hatchability) were assessed using fixed experimental parameters (distance from light source to target, 150 mm; total target surface area, 2.16 cm^2^; the power output could be adjusted to up to 300 mW). The exposure time for the mites and eggs varied between 1 and 4 min depending on the repetition rate of the laser pulses.

## Methods

### Pulsed UV-C non-ionizing irradiation system

**Fig. 1:**
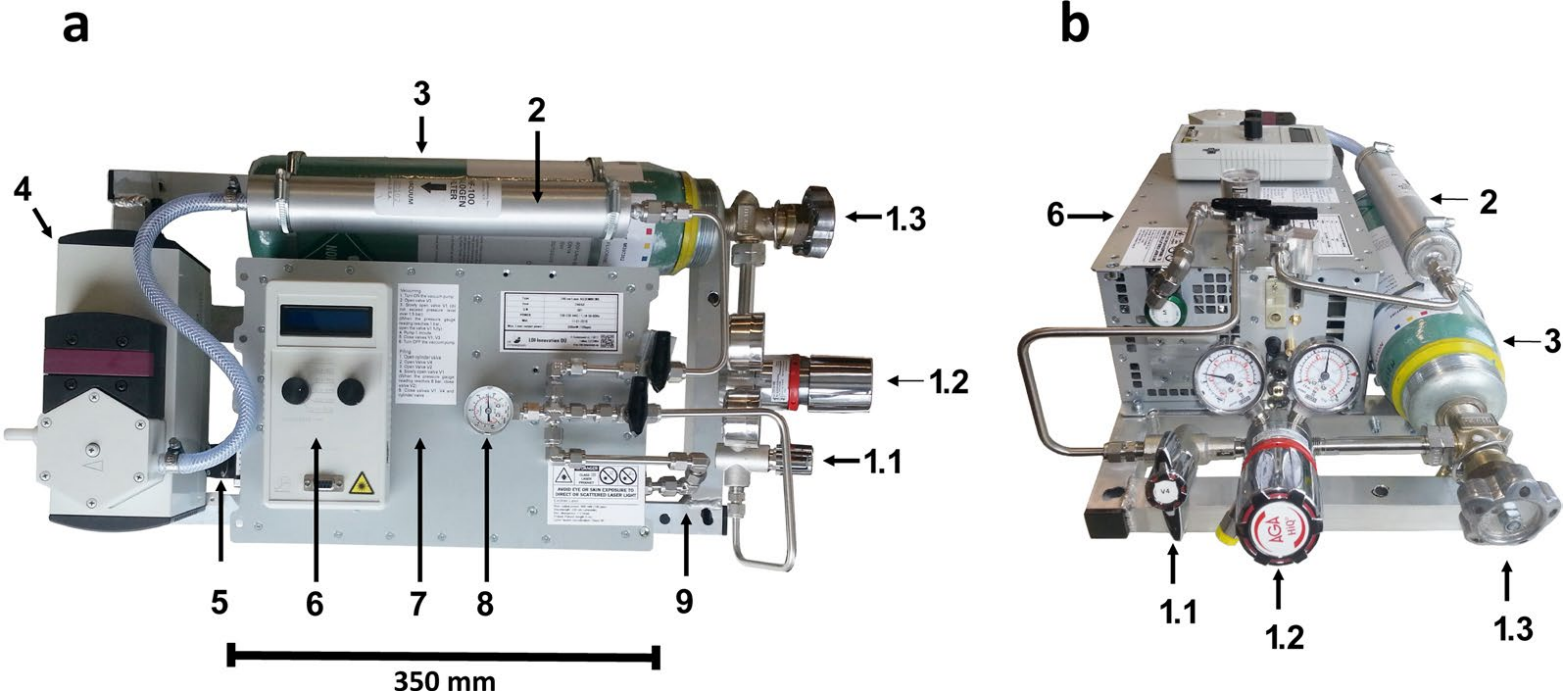
Top (**a**) and front (**b**) views of the ultraviolet (UV)-C generator components. **a**, **b** Valve for connection to the laser gas system (*1.1*), pressure reducer valve (*1.2*), excimer gas cylinder valve (*1.3*), halogen filter (*2*), krypton fluoride (KrF) gas refill cylinder (*3*), energy meter display (*6*). **a** Vacuum pump (*4*), optical system (*5*), excimer laser CEX-100 (*7*), pressure gauge (*8*), mechanical frame (*9*)

The UV-C radiation-dose system comprises several interconnected modules designed in Estonia by LDI Innovation OÜ (www.ldi-innovation.com) for the current study. These include the proprietary excimer laser CEX-100 with gas refill module, the beam shaping and beam delivery module, and the laser meter used to assess the radiation levels at the target site (Fig. 1) (Additional file 1: Figure S1). This system is piloted by embedded computer software for the control of laser frequency and exposure duration, and for automatic calculation of the UV dose received by the target according to the specified beam size and beam power.

The laser source was the CEX-100 KrF excimer laser, manufactured in Estonia by LDI Innovation OÜ. The excimer laser operates at 248 nm (FWHM < 1 nm) emission wavelength with more than 5 mJ of pulse at ~ 10-ns pulse duration with a pulse repetition rate of up to 60 Hz (Additional file 2: Figure S2), and has a 120-W electrical power input. The laser-based radiation-dose system requires a built-in excimer container, which serves as the gas-refilling source.

To monitor the refilling procedure, the KrF gas cylinder is equipped with a valve, a pressure gauge, a halogen filter for exhaust gas safety, and a vacuum pump to enable the extraction of exhaust gases from the laser chamber. Continuous operation requires a gas refill every 2 weeks. The refill procedure was done manually due to the frequent use of the system in the experiment. The refill procedure takes less than 10 min, during which time the laser is disabled. The refill procedure is fully automated for operational use, which allows for more than a year of maintenance-free operation until the gas cylinder is empty.

The beam delivery and shaping module is equipped with a variety of cut-off apertures (1:1, 1:2, 1:6 and 1:10 of the initial beam power) and a removable fused silica lens which is used to diverge the beam. The latter enables divergence of the laser beam to allow the relatively even distribution of laser energy on larger surface areas in a controlled and measurable manner. Both the laser and gas modules, and the laser energy meter, are installed on a mechanical frame.

The laser power meter is based on a National Institute of Standards and Technology calibrated GreenTEG B05 thermopile power sensor, the output of which is amplified and displayed on an LCD screen. The power meter components are the measurement head, which is positioned under the laser beam, and the display. The energy levels that are delivered to the target are calculated by the included software. The latter enables control of the laser frequency and exposure time, and automatically calculates target-delivered UV doses for a pre-set beam size and beam power.

### Exposure set-up

**Fig. 2:**
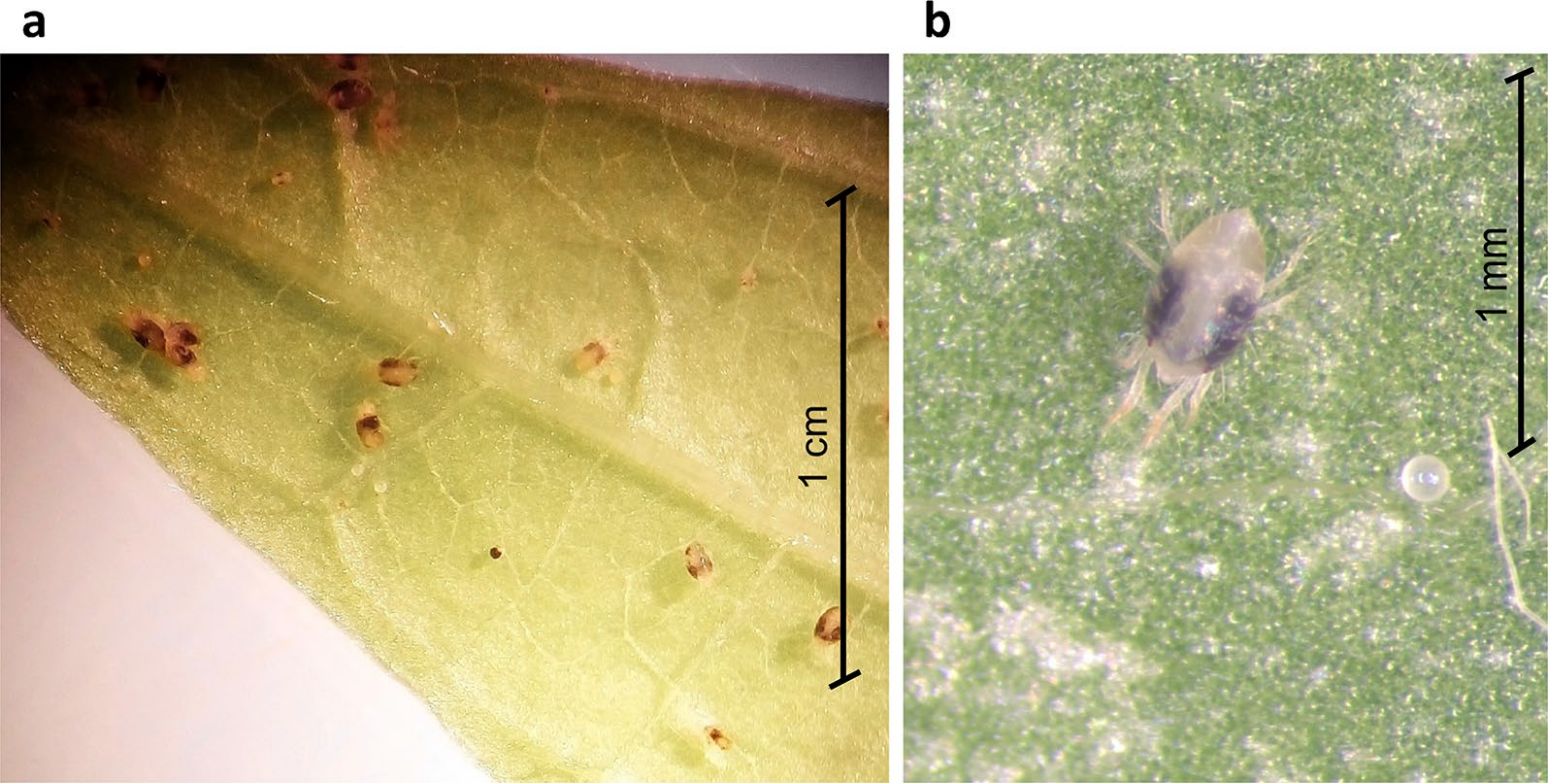
Two-spotted spider mites *Tetranychus urticae* (**a**) and an adult female and egg (**b**) on a common bean (*Phaseolus vulgaris*) leaf

*Tetranychus urticae* were collected from infested plants in citrus orchards in Tunisia and transferred to a climate room (26 °C, 50–60% relative humidity, 16:8-h light:dark) in our laboratory at the Biodiversity Research Centre, Université catholique de Louvain, Louvain-la-Neuve, Belgium. The mites had not been exposed to any acaricides before the experiments. The *T. urticae* were reared on the leaves of the common bean (*Phaseolus vulgaris*) placed on moistened cotton in Petri dishes [27]. Individuals of known, synchronized age were obtained by allowing adult females to lay eggs for a maximum of 24 h on a leaf (Fig. 2). After this time, the females were removed and the eggs continued to develop to the desired stage.

**Fig. 3:**
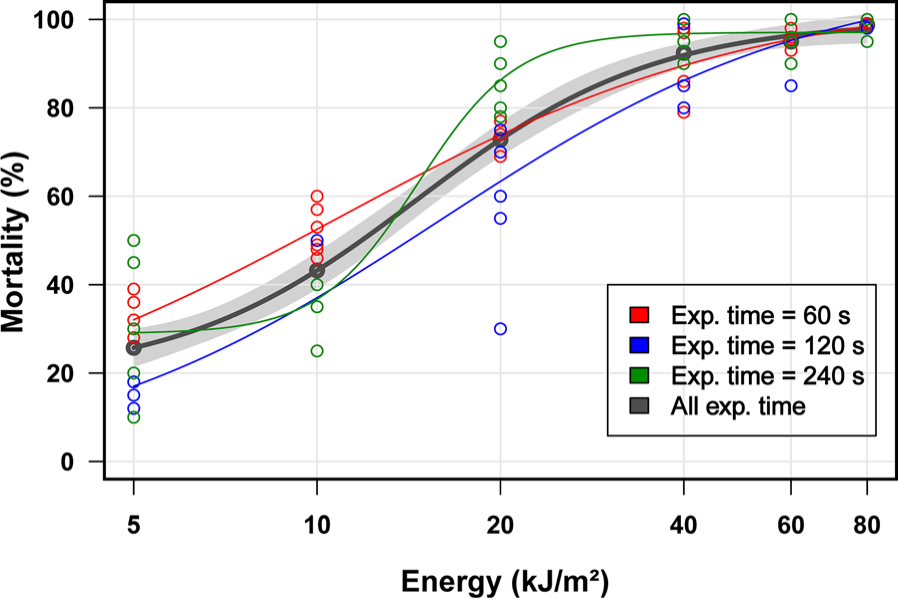
Relation between the energy delivered to the adult female mites and observed mortality after irradiation at three different exposure times (60, 120, and 240 s) using the pulsed KrF excimer laser; the study was undertaken at 20–25 °C, in April 2018. Average mortality curve (*thick black line*; calculated using data for the entire exposure period) and corresponding confidence interval (*light grey band*). Parameter estimates for the four-parameter log-logistic regression model are as follows: slope − 2.22, lower limit 18.87, upper limit 99.69, median effective dose 14.60

 For each experiment, an average of ten adult female *T. urticae* were collected from the bean leaves by using a camel hair brush under a stereomicroscope. The females were then transferred to a section of leaf in an aluminium chamber that had been placed in a Petri dish in such a way as to flatten the leaf surface to prevent the two-spotted spider mites from escaping the radiation beam (Petri dish inner diameter, 35 mm; inner dimensions of the rectangular chamber illuminated by laser light, 12 mm × 18 mm × 2 mm) (Fig. 3) (Additional file 3: Figure S3). Only young adult (24 h old) females were used in the bioassay (Additional file 4: Figure S4).

### Survival study of mites and eggs after UV irradiation

The effect of UV irradiation on mite mortality was assessed using adult female mites. For each laser exposure condition, the mortality rate was calculated as the ratio of the number of dead mites to the total number (*n* ~ 10) of mites used. The study consisted of five independent experiments with fixed and variable parameters. The fixed parameters were the distance from the light source to the target (150 mm), which resulted in a total target surface area of 2.16 cm^2^, and the laser pulse energy (5 mJ). The variable parameters included the three exposure durations (60, 120 and 240 s) and multiple laser frequency settings, which ranged from 4 to 60 Hz for 60 s, 2–30 Hz for 120 s, and 1–15 Hz for 240 s. For each exposure duration, the combination of both variable parameters resulted in six different energy doses per surface area (5, 10, 20, 40, 60, 80 kJ/m^2^).

Mortality curves were computed using R.4.1.1 and the dcr R package (version 3.0.1), which is used for dose–response analysis [31]. The drm function of this package was used to build a four-parameter log-logistic model. The resulting mortalities are reported as three distinct curves for the exposure durations and mortalities per surface energy density. A curve of averaged mortality (based on data for all exposure durations) and the corresponding 95% confidence interval are also reported.

The lethal effect of the UV-C irradiation was then assessed for eggs at 24 h post-exposure by direct observation of egg hatching, using the same experimental design, and up to 12 days post-irradiation. Mortality curves were computed for the exposed and unexposed eggs using the Loess function in R.4.1.1; the unexposed eggs were used as the control. For the control, a section of leaf with a similar surface area to that on which the eggs had been placed was put on the top of the eggs to protect them from the UV-C irradiation.

## Results

The immediate microscopic post-irradiation assessment showed visible effects of UV-C radiation on the adult female mites, as many of them had stopped moving. The effect of UV-C irradiation of the adult female mites was quantified after 24 h, and showed that they were significantly impacted by the amount of energy delivered. The average mortality (i.e. the fitted value of the averaged results; Fig. 3) was 26% and 92% at 5 and 40 kJ/m^2^, respectively. Mortality at 80 kJ/m^2^ reached 98%. The effect of exposure duration on mortality was minimal, as depicted by the superposition of the three curves corresponding to the different exposure times. The results showed that a reasonably high level of mortality could already be achieved after less than 1 min of exposure to the UV-C radiation produced by the laser beam.

**Fig. 4:**
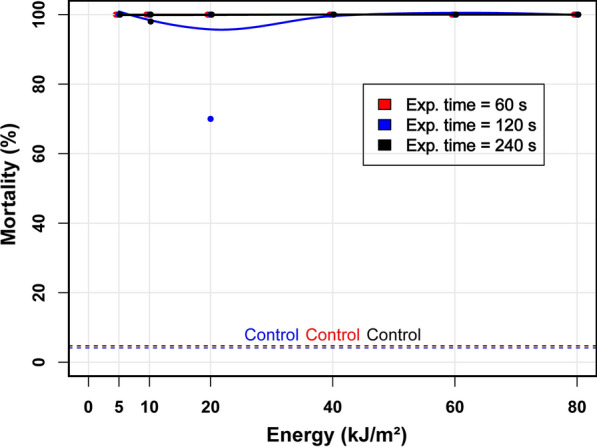
Relation between the energy delivered to the mite eggs and egg mortality under three radiation exposure times (60, 120, and 240 s) using the pulsed KrF excimer gas laser; the study was undertaken at 20–25 °C, in April 2018.* Dashed line* indicates mortality for the controls, which were not exposed to UV-C radiation

The effect of irradiation on the mortality of eggs was even more dramatic, as this reached almost 100% regardless of the delivered energy dose (5–80 kJ/m^2^) for each exposure time (Fig. 4), indicating that even the lowest energy density exceeded that for egg viability. After UV-C irradiation, the eggs conserved their shape for the first few days but then turned progressively yellow, dried out and collapsed (Additional file 5: Figure S5). There was no observed effect of UV-C irradiation on the leaves.

## Discussion

GUVI is a disinfection method that is used to inactivate and kill microorganisms as a direct consequence of their exposure to UV radiation and absorption of high energy photons, especially at short-wavelength UV-C (200–280 nm), a range of wavelengths known to be germicidal. The rationale for using a wavelength of 248 nm in the current study is that nucleic acids show their highest light absorbance at around this wavelength [7]. The mechanisms of cell alteration following exposure to high energy photons are manifold. Firstly, UV-C irradiation causes photobiologic effects that are proportional to the duration and repetition of light exposure, i.e. to the amount of energy delivered to uni- and multicellular organisms [32]. When all the photon energy is transferred to the atoms and molecules that absorb it, the resulting excited states may lead to protein alteration and enzyme inactivation, which may hamper key biologic systems, e.g. through the splitting of peptide bonds, the alteration of sulfide and disulfide bonds, and photochemical oxidation [33, 34]. Cyclobutane pyrimidine dimers, which are formed between two adjacent pyrimidines in the same strand of DNA by the cross-linking of cytosine and thymine, are considered the most important products of UV-induced damage to the DNA; they cause local denaturation of the DNA helix, prevent DNA repair, inhibit DNA replication and protein synthesis, damage RNA, lead to the loss of essential metabolic activities and cause cellular death [7, 8]. Photons emitted by UV-C may induce oxidative processes through photooxidative reactions, depending on the number of incident photons and those absorbed by molecules, which varies according to the structure of the latter [7].

Over the last two decades, many studies on the effects of UV radiation on *T. urticae* have been published, though data on the effect of UV-C radiation emitted by a laser excimer on *T. urticae* mortality are scarce, and none of those studies used a KrF laser excimer [30, 35–47]. A recent review examined the biological impact of UV-B radiation (280–315 nm), but not UV-C radiation, on spider mite populations [48]. However, the review did note the sensitivity of spider mites to UV radiation and that *T. urticae* mostly remain on the underside of leaves as a UV-avoidance mechanism, except during diapause, when they appear to be better protected from UV radiation by synthesising keto-carotenoids, and notably astaxanthin. Using a 300-W Xenon light source with band-pass filters, Sakai and Osakabe [49] exposed eggs of *T. urticae* to UV radiation at wavelengths of 280–320 nm for 1 h; they showed that the eggs exposed to 280–300 nm radiation never hatched, while hatchability was not affected at the other wavelengths. In the present study, the eggs of *T. urticae* were particularly susceptible to the treatments, as 100% mortality was observed. In a recent study, Short et al. [29] used UV-C irradiation to control *T. urticae* on whole strawberry plants in small phytotrons. Infested plants that underwent nightly 60-s exposure to low doses of UV radiation (1.2 W/m^2^) showed a 97% decrease in *T. urticae* over the entire plant, and a 99% decrease in the middle and upper portions of the plants compared to the untreated plants. No phytotoxic effect was observed. Moreover, the UV-C-irradiated plants were not covered with spider mite webs, whereas 65% of the control plants were. Albeit obtained using a different technological set-up to ours, Short et al.’s [29] results on the effects of UV-C radiation (254 nm) on *T. urticae* corroborate our findings, and clearly show the potential of this new technology, even if additional studies on the mechanisms of action and the conditions of implementation are still needed [29]. It is also clear that mite mortality and lethal egg damage are proportional to the total amount of UV-C energy delivered in 1 min. Similar results showing a clear increase in mortality that was proportional to time of exposure and proximity to the radiation source were obtained for the dust mites *Dermatophagoides pteronyssinus* and *Dermatophagoides farinae* when using a 88-cm × 2.5-cm, 30-W, UV germicidal lamp that emitted radiation in the UV-C range at 254 nm, but for a longer duration, i.e. 5–60 min [50]. Lah et al. [50] observed 100% mortality after 60 min at a 12-cm distance, while egg mortality was 100% irrespective of the distance and time of exposure compared to 70% hatchability of the eggs used as the control. Suzuki et al. [30] reported a similar response to UV-C radiation in *T. urticae*. The ~ 100% mortality of the female adults and eggs of the two-spotted spider mite *T. urticae* after less than 1 min of exposure to UV-C pulsed irradiation in our study compares favourably to previous data.

With the 2 × 3.5-milliradian divergence of its top-hat profiled rectangular laser beam defining the smallest spot size for long-distance applications, the experimental UV-C generator with a pulsed KrF excimer laser used in the present study has a number of key features, such as output modulation, applicability to smaller or larger spots, and long-distance application whenever needed. These features increase the potential application of this experimental UV-C generator with a pulsed KrF excimer laser compared to other UV-C radiation tools [51, 52], and indeed open up new control possibilities in areas as varied as food infestation by mites and combatting the poultry red mite (*Dermanyssus gallinae*). Despite the challenges associated with radiation efficacy and the size of the surface which needs to be decontaminated, the capacity to deliver UV-C radiation over a long distance is mandatory for this type of application. With accurate beam shaping over a long distance, a lethal dose can be directed onto mite eggs by just a few pulses (less than 5 kJ/m^2^ is required for the eggs of *T. urticae*) and even onto moving mites before they can escape to a protected area such as the underside of the leaf; however, the effect of this type of escape requires further investigation and additional data. Furthermore, choosing a robust excimer laser gives researchers the flexibility to use this type of laser system in experiments in most industrial settings, without potential issues in terms of vibration or temperature conditions, though potential synergistic associations with other decontaminating thermal and non-thermal treatments should be born in mind [53], as should the potential occurrence of UV-C-induced resistance among microbial agents. For the purpose of thoroughness, it should be mentioned that the lethal dose of UV-C radiation for plant matter is substantially higher than the values obtained here for adult mites [54]. This enables the effective and safe eradication of mite eggs while using active beam steering and focusing to kill adult mites, and ensures that the UV-C irradiation of *T. urticae* adults and eggs with a pulsed KrF excimer laser is applicable to real-life situations.

## Conclusions

The KrF excimer laser used here is a robust and relatively cheap high-intensity deep-UV light source. As shown in this study using *T. urticae*, the laser beam shaping achieved with this KrF excimer enables the targetting of a precise area contaminated with eukaryotic, or prokaryotic, organisms (e.g. pests and biothreats) with UV radiation to kill them. Using a laser as the source of UV light enables repeatable beam shaping and the application of UV radiation over a long range, and also allows for the comparison of UV-induced damage with that induced thermally. However, the use of this system for bio-control applications for distant and large surfaces requires further research and development to better understand the mechanisms involved, and to determine the best techniques and optimal set-up for its application. In future work, the focus also needs to be on the germicidal effects of UV-C produced by this generator for a range of microbial pathogens by using various matrices. The field of application needs to be refined and potential limitations (such as UV-C-associated health risks) examined. Furthermore, the area that can be exposed to the UV radiation produced needs to be extended and controlled, and the ability of laser systems to irradiate targets in real-life conditions over a long distance whilst integrated into portable handheld systems also needs to be investigated.

## Supplementary Information

**Additional file 1: Figure S1.** Three-dimensional computed views of the pulsed UV-C non-ionizing irradiation system. The following device components are shown: 248-nm laser (*grey*,* dark grey* and* violet*), vacuum pump (*light grey*), gas cylinder (*green*), gas filter (*red*), power meter (*yellow*), Petri dish (*light green*), optical module (*white*) with shutter (*red*), frame with adjustable legs (*dashed white line*).

**Additional file 2: Figure S2.** Video showing pulsed UV-C laser beam.

**Additional file 3: Figure S3.** Experimental set-up used to irradiate the two-spotted spider mites. Petri dish (**a**), aluminium chamber (**b**), and aluminium chamber inserted into the Petri dish (**c**).

**Additional file 4: Figure S4.** Video showing live two-spotted mites and egg on a leaf.

**Additional file 5: Figure S5.** Microscopic appearance of *Tetranychus urticae* eggs 9 and 12 days after pulsed UV-C irradiation (accumulated dose of 5 kJ/m^2^ over 60 s) generated by the pulsed krypton fluoride excimer laser. The appearance and colour of eggs at 9 days (**a**) and 12 days (**b**) post-irradiation.

## Data Availability

The datasets supporting the findings of this article are included within the paper.

## Abbreviations

FWHM: Full width at half maximum
GUVI: Germicidal ultraviolet irradiation
KrF: Krypton fluoride
LED: Light-emitting diode
UV: Ultraviolet
