# Supplementary figures and images for: Acaricidal efficacy of ultraviolet-C irradiation of Tetranychus urticae adults and eggs using a pulsed krypton fluoride excimer laser
Source: Parasit Vectors. 2021 Nov 17;14:578. doi: 10.1186/s13071-021-05085-7 (PMC8596343; doi:10.1186/s13071-021-05085-7)

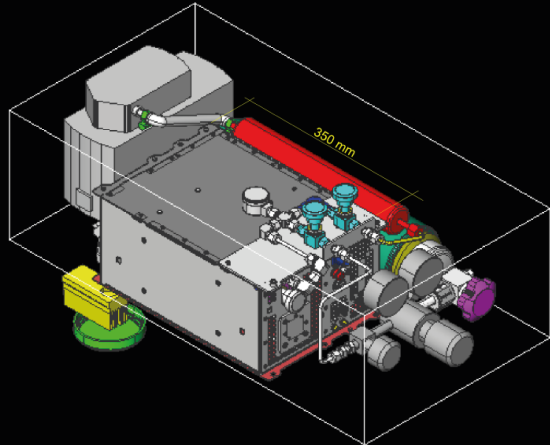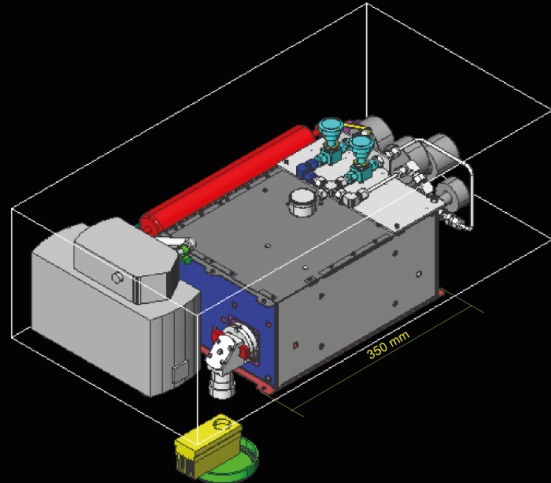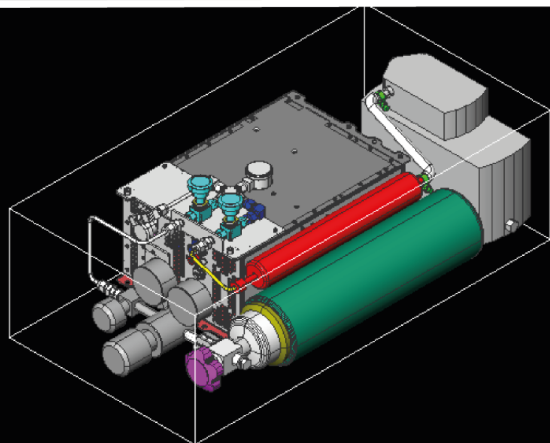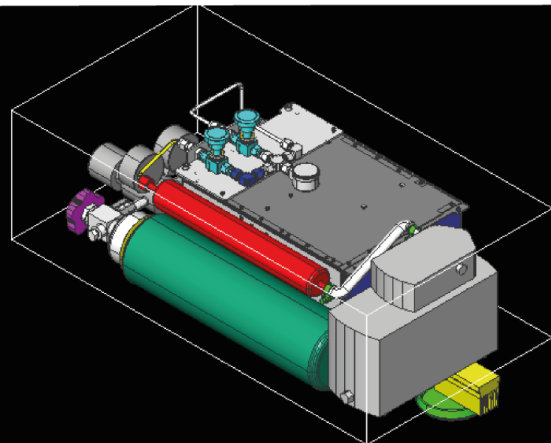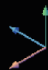

Supplement: Supplementary file 1 — Additional file 1: Figure S1. Three-dimensional computed views of the pulsed UV-C non-ionizing irradiation system. The following device components are shown: 248-nm laser (grey, dark grey and violet), vacuum pump (light grey), gas cylinder (green), gas filter (red), power meter (yellow), Petri dish (light green), optical module (white) with shutter (red), frame with adjustable legs (dashed white line). [file 13071_2021_5085_MOESM1_ESM.pdf]

**a**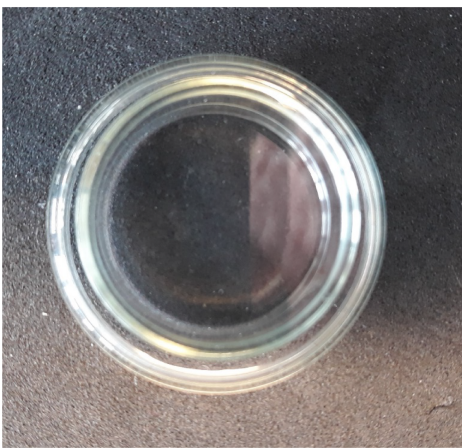**b**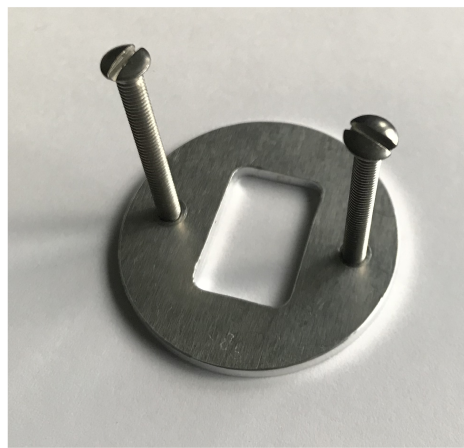**c**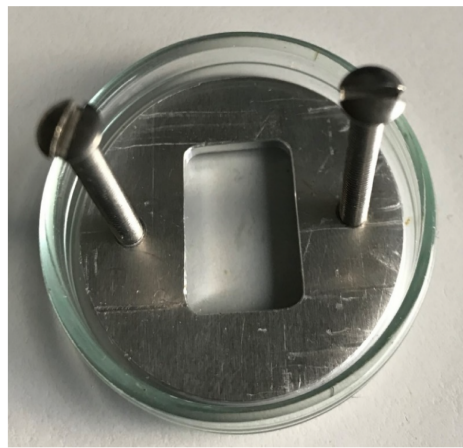

Supplement: Supplementary file 3 — Additional file 3: Figure S3. Experimental set-up used to irradiate the two-spotted spider mites. Petri dish (a), aluminium chamber (b), and aluminium chamber inserted into the Petri dish (c). [file 13071_2021_5085_MOESM3_ESM.pdf]

**a**

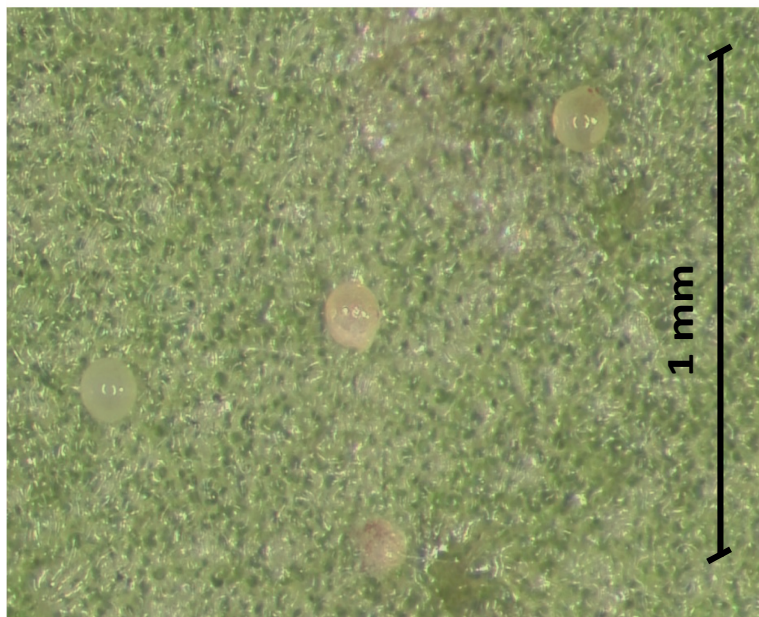

**b**

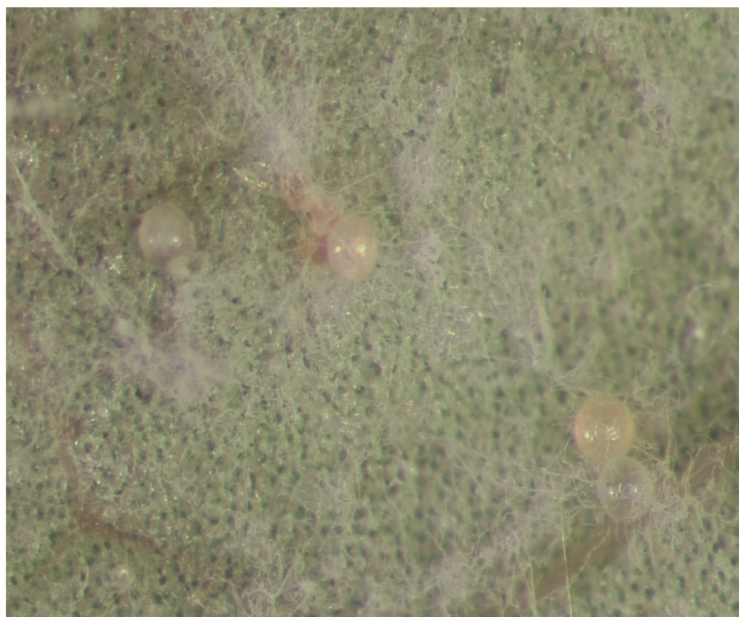

Supplement: Supplementary file 5 — Additional file 5: Figure S5. Microscopic appearance of Tetranychus urticae eggs 9 and 12 days after pulsed UV-C irradiation (accumulated dose of 5 kJ/m2 over 60 s) generated by the pulsed krypton fluoride excimer laser. The appearance and colour of eggs at 9 days (a) and 12 days (b) post-irradiation. [file 13071_2021_5085_MOESM5_ESM.pdf]
